# Diversity of staphylococcal cassette chromosome *mec* elements in nosocomial multiresistant *Staphylococcus haemolyticus* isolates

**DOI:** 10.1007/s13353-016-0346-5

**Published:** 2016-04-07

**Authors:** Ewa Szczuka, Magdalena Krajewska, Dagmara Lijewska, Karolina Bosacka, Adam Kaznowski

**Affiliations:** 1 Department of Microbiology, Institute of Experimental Biology, Faculty of Biology, Adam Mickiewicz University, ul. Umultowska 89, Poznań, 61-614 Poland; 2 Department of Microbiological and Laboratory Diagnostics, Bacteriological Laboratory, Regional Hospital in Poznań, Juraszów 7/19, Poznań, 60-479 Poland

**Keywords:** *Staphylococcus haemolyticus*, SCC*mec*, Antibiotic resistance

## Abstract

*Staphylococcus haemolyticus* is the second, most frequently isolated coagulase-negative staphyloccus (CoNS) from patients with hospital-acquired infections, and it is usually resistant to methicillin and other semisynthetic penicillins. The purpose of this study was to characterize staphylococcal cassette chromosome *mec* (SCC*mec*) elements and assess the in-vitro activity of antibiotics against 60 *S. haemolyticus* strains recovered from hospitalized patients. All these strains expressed methicillin resistance and carried a *mecA* gene. Moreover, all strains possessed a multiresistant phenotype, i.e., exhibited resistance to more than three classes of antibiotics. Eleven strains (18 %) harbored the SCC*mec* type V, containing *ccrC* and *mec* complex *C*. Three isolates harboring the *ccrC* gene did not contain a known *mec* complex. One strain positive for *mec* complex C was not typeable for *ccr*. This suggests that *ccrC* and *mec* complex *C* may exist autonomously. Only four strains carried *mec* complex B, whereas none of the *S. haemolyticus* harboured *mec* complex A. A new combination, which is *mec* complex B-*ccrAB*_ship_, was found in *S. haemolitycus*. The *ccrAB*_ship_ was also identified in two strains of *S. haemolitycus* in which the *mec* gene complex was not identified. The results of the present study indicate that in *S. haemolyticus* the *mec* gene complex and the *ccr* genes are highly divergent. However, *ccr* sequence analysis does not allow the identification of a new allotype, based on a cut-off value of 85 % identity. The *ccr* genes in the *S. haemolitycus* strain showed ≥96 % sequence identity to the *ccrAB2* genes.

## Introduction

*Staphylococcus haemolyticus* belongs to the group of coagulase-negative staphylococci (CoNS) and is a part of the normal skin flora and mucous membranes. It is an opportunistic pathogen capable of causing various types of infections, including bacteremia, meningitis, skin infection, prosthetic joint infections, and endocarditis, associated with adherence of staphylococci to medical devices and ability of these bacteria to biofilm formation (Götz et al. 2006; Kristóf et al. 2011; Nunes et al. 2005; Rodhe et al. 2006; Szczuka et al. 2015). Other virulence factors, such as exotoxins and enzymes, including nucleases, proteases, lipases, and hemolysins as well as cytotoxic activity through induction of apoptosis, also play a role in the pathogenesis of this species (Kloos and Bannerman 1999; Krzymińska et al. 2012, 2015). Among CoNS, *S. haemolyticus* has the highest tendency for developing resistance to multiple antibiotics, and it is usually resistant to methicillin and other semisynthetic penicillins (Barros et al. 2012; Krediet et al. 2001, 2004). The methicillin resistance of staphylococci is based on the expression of a modified penicillin-binding protein transpeptidase, with a low affinity for ß-lactams, known as PBP2a or PBP2′, which is encoded by the *mec*A gene. Notably, a novel PBP2a homolog was described as being encoded by *mecC*, which shares 70 % identity with *mecA* at the DNA level. The *mecC* conferred cefoxitin and oxacillin resistance in *S. aureus* strains (Ballhausen et al. 2014). The *mec*A gene is carried on a mobile genetic element, called the staphylococcal chromosome *mec* (SCC*mec*) integrated into the chromosome at a specific site, located near the origin of replication. To date, eleven SCC*mec* types have been assigned for *Staphylococcus aureus* based on the composition of the *ccr* gene complex and the class of the *mec* gene complex (Ito et al. 2001, 2004; IWG-SCC 2009). The *mec* gene complex (A-E) is composed of a *mecA* gene, regulatory genes (*mecRI* and *mecI*), a hypervariable region (HVR), and an associated insertion sequence. The *ccr* gene complex (type *ccrA*, *ccrB* and *ccrC*) contains recombinase genes encoding recombinases responsible for the precise excision and integration of *SCCmec* within the bacterial chromosome. The *ccrA* and *ccrB* genes have been classified into different allotypes, whereas there is only one *ccrC* allotype (IWG-SCC 2009). Extra *ccr* allotypes have been also identified, i.e., *ccrA5* in *S. pseudintermedius*, *ccrB6* in *S. saprophyticus*, *ccrB7* in *S. saprophyticus, ccrA5* in *S. cohnii*, and *ccrAB*_ship_ in *S. haemolyticus* (IWG 2009; Pi et al. 2009; Zong and Lü 2010). The *ccrAB*_ship_ products catalyse the mobility of SCC*mec* and might be responsible for movement of the arginine catabolic mobile element (ACME). This element carries genes encoding a arginine deiminase pathway that converts L-arginine to carbon dioxide, ATP, and ammonia. This metabolic pathway is important for bacteria survival at low pH and for inhibition of the immune response against bacterial infections (Pi et al. 2009). In addition to the *ccr* and *mec* gene complex, the SCC*mec* element contains various other mobile genetic elements (MGE), e.g., plasmid, insertion sequence, and transposon mediating resistance to non-β-lactam antibiotics or heavy metals (Shore and Coleman 2013). It is believed that the increase in resistance of staphylococci to antibiotics is in part due to the presence of the SCC*mec*, which could be easily transferred between staphylococcal species (Hanssen and Ericson Sollid 2006). The horizontal transfer of a SCC*mec* type V from MRSH (methicillin-resistant *S. haemolyticus*) to methicillin-susceptible *S. aureus* strains resulted in creating MRSA clone (Berglund and Söderquist 2008).

Currently, there is great concern given to the increasing number of methicillin-resistant *S. haemolyticus* (MRSH) strains that are able to cause severe infections in hospitalized people. These bacteria show a remarkable tendency of developing resistance to multiple antibiotics, as well as the potential to transfer SCC*mec* elements. This study was undertaken to characterize the staphylococcal cassette chromosome in clinical MRSH isolates, and determine the susceptibility profiles of these isolates.

## Material and methods

### Bacterial strains

Sixty isolates of *S. haemolyticus* were collected from clinical specimens of patients treated at the Regional Hospital in Poznań. Only isolates considered clinically relevant were included in this study. Isolates were identified by Gram staining, colony morphology, catalase reaction and by biochemical tests using the a Vitek 2 system (bioMérieux, France). The isolates were stored at in medium with 25 % glycerol at −70^o^ C.

### Susceptibility testing

Resistance to β-lactams was determined by the cefoxitin (30 μg) screen test as well as by amplification of *mecA* gene by PCR technique. Analysis of susceptibility to the following antibiotical agents was also performed using a Vitek 2 system (bioMérieux, France) according to EUCAST recommendations (http://www.eucast.org/clinical_breakpoints): fluoroquinolones (ciprofloxacin, levofloxacin, moxifloxacin), aminoglycosides (gentamicin and tobramycin), glycopeptides (teicoplanin and vancomycin), macrolides and lincosamides (clindamycin and erythromycin), tetracyclines (tetracycline and tigecycline), and others (linezolid, rifampin, trimethoprim-sulfamethoxazole, fusidic acid and fosfomycin) .

### Preparation of total DNA for PCR

The total DNA was isolated and purified using the Genomic Mini DNA kit (A&A Biotechnology, Gdynia, Poland).

### SCC*mec* analysis

The detection of *mecA* gene was carried out using primers and method as described previously (Zhang et al. 2005). The SCC*mec* analysis was carried out by identification of the *mec* complex and *crr* genes by PCR method according to the criteria set out for *S. aureus* (Zhang et al. 2005). In addition, the detection of gene lineages of *mecA-mecI* (class A *mec*), *mecA- IS1272* (class B *mec*), and *mecA- IS431* (class C *mec*) was performed by PCR technique using four primers assigned by Kondo et al. (2007). The presence of the novel *ccr* allotype (*ccrAB*_SHP_) in *S. haemolyticus* isolates was checked with primers assigned by Pi et al. (2009). The amplification products were electrophoresed in 1.5 % agarose gel. The gels were stained with ethidium bromide, visualized on a UV light transilluminator, and documented with a V.99 Bio-Print system (Vilber Lourmat, Torcy, France).

## Sequence analysis

The *ccrA* and *ccrB* genes amplicons were obtained using primers designed by Zong and Lü (2010). PCR products were purified and sequenced in a 3100xl Genetic Analyzer (Applied Biosystems). The resulting sequences were deposited in GenBank with accession numbers KU523873 and KU523874. These sequences were compared with *ccr* allotypes, available in GenBank data: *ccrA1* (*S. aureus* 45394 F, *S. hominis* GIFU12263), *ccrA2* (*S. aureus* M06/0075, *S. aureus* JCSC6668, *S. aureus* N315), *ccrA3* (*S. aureus* JKD608, *S. pseudintermedius* KM1381), *ccrA4-2* (*S. aureus* CHE482), *ccrA4-1* (*S. aureus* CHE482), *ccrA5* (*S. pseudintermedius* KM241), *ccrA*_SHP_ (*S haemolyticus* strain H9), *ccrB1* (*S. aureus* COL), *ccrB2* (*S. aureus* JCSC1968, *S. epidermidis* CS8), *ccrB3* (*S. aureus* TW20, *S. pseudintermedius* KM1381, *S. cohnii* WC28), *ccrB4* (*S aureus* BK20781, *S. aureus* HDE288, *S aureus* strain CHE482), *ccrB5* (*S. pseudintermedius* KM241), and *ccrB*_SHP_ (*S haemolyticus* H9). Similarity searches were carried out using BLAST programs (http://www.ncbi.nlm.nih.gov/BLAST/).

## Results and discussion

The *S. haemolyticus* strains included in this study originated from hospitalized patients with nosocomial infections. All of these isolates expressed phenotype MR and carried a *mecA* gene. Moreover, all isolates possessed a multiresistant phenotype, i.e., exhibited resistance to at least three of the non-ß-lactam antibiotics tested. The majority of MRSH isolates were resistant to erythromycin (95 %) and gentamicin (90 %); fewer isolates were resistant to clindamycin (77 %), trimethoprim-sulfamethoxazole (73 %), ciprofloxacin (58 %), tetracycline (34 %), tobramycin (27 %), and levofloxacin (18 %). Only single strains were resistant to moxifloxacin, rifampin, fusidic acid, tigecycline, and fosfomycin. Resistance among *S. haemolyticus* strains isolated from clinical specimens underscores the importance of this species as a reservoir for *mecA* genes (Nunes et al. 2005; Tabe et al. 2001). The resistance to a great range of antibiotic agents is thought to be associated with the existence of many insertion sequences, which accumulate antibiotic resistance genes, in the *S. haemolyticus* genome (Takeuchi et al. 2005). It should be underlined that all isolates were susceptible to vancomycin and a relatively new antibiotic — linezolid. Recently, linezolid-resistant *S. haemolyticus* has been described in European countries, the USA, China, and India (Cai et al. 2012; Gupta et al. 2012; Mazzariol et al. 2012; Rodríguez-Aranda et al. 2009; Tewhey et al. 2014).

The SSC*mec* type V, containing *ccrC* and *mec* complex C, was detected in 11 out of 60 *S. haemolyticus* tested strains (18 %) (Table 1). The results of this study are similar to those of Ruppe et al. (2009), who demonstrated that SCC*mec* type V is preferentially associated with *S. haemolyticus* strains isolated from distinct geographical areas, such as Cambodia, Algeria, Mali, and Moldova. Data obtained by Bouchami et al. (2012) indicated that *ccrC* and *mec* complex C are most prevalent among *S. haemolyticus*. It was also found that the *ccrC* and *mec complex* C may exist autonomously. In our study, the *ccrC* alone was observed in three *S. haemolyticus* strains. One strain was only positive for *mec* complex C. Forty-nine isolates (82 %) were non-typeable for *SCCmec* using currently-available schemas based on the multiple PCR method. This can be explained by the presence of novel structures or frequent genomic rearrangements and recombination of the SCC*mec* element. It is noteworthy that the *SCCmec* types II, III and V have been detected in *S. haemolyticus* collected in China (Zong et al. 2011). It is believed that occurrence of different SCC*mec* types in China might reflect the genetic background of *S. haemolyticus* strains, connected with geographical locations. None of the studied isolates harboured the *mec* complex A. The four *S. haemolyticus* strains appeared to carry the class B *mec*. For two of these strains the c*rr* gene could not be amplified. It should be noted that the class B *mec* complex, as well as the *ccr* type 2 (SCC*mec* type IV), was described in a MRSH strains collected in Norway (Ibrahem et al. 2009). In summary, most *S. haemolyticus* strains did not exhibit a known *mec* complex. Similarly, Bouchami et al. (2012) did not detect *mec* gene complex in half of the MRSH isolates. In another report, in 15 *S. haemolyticus* strains the *mec* complex was also non-typeable (Garza-González et al. 2010).

**Table 1:** SCC*mec* typing results

No. of isolates	SCC*mec* type	Origin	*mec* class	*ccr* type
11	Type V	blood, endotracheal aspirate, wound secretion, urine, fluid from the peritoneal cavity	class C	*ccr*C
3	NT	blood	ND	*ccr*C
1	NT	blood	class C	ND
2	NT	blood, synovial fluid	class B	*ccrAB*_ship_
2	NT	blood, endotracheal aspirate	ND	*ccrAB*_ship_
2	NT	blood, endotracheal aspirate	class B	ND
39	NT	blood, endotracheal aspirate, wound secretion, urine, fluid from the peritoneal cavity, venous catheter	ND	ND

*ND*, not detected

*NT*, untypable

As we mentioned above, fourteen isolates carried *ccrC*. Additionally, four isolates exhibited the *ccrAB*_ship_ allotype. The results of this study are similar to reports by Pi et al. (2009), who detected *ccrAB*_ship_ genes in eight *S. haemolyticus* strains. These *ccrAB*_ship_-positive strains carried the class C *mec* complex. Among *S. haemolyticus* strains studied by us, two with the *ccrAB*_ship_ allotype lacked a known *mec* complex, while the two remaining *ccrAB*_ship_–positive strains carried the *mec* complex B. To our knowledge, this is the first time that a unique combination of the *ccr* gene complex and the *mec* gene complex (*mec* complex B/*ccrAB*_ship_) has been identified in staphylococcal strains. It should be emphasised that in 42 isolates the *ccr* gene was not identified. We cannot exclude the loss of the *ccr* complex from the SCC*mec* element. Recently, Zong (2013) found that the *mecA* gene may exist without *ccr* genes, and he suggested that the *mecA* gene, bracketed by two copies of IS431 forming a composite transposon, could be transferred. The failure to detect *ccr* in *S. haemolyticus* strains may be explained by the fact that they represent novel allotypes of recombinase. Therefore, newly-designed primers pairs were used to amplify *ccrA* and *ccrB* genes and the sequences of obtained amplicons were determined, deposited in GenBank with accession numbers KU523873 and KU523874, and compared to known *ccr* sequences from GenBank database. The *ccrA* gene (KU523874) that was identified in a *S. haemolyticus* isolate (MPU SH 68) displayed ≥96 % sequence similarity to the *ccrA2* gene of the *Staphylococcus* aureus strains (M06/0075, JCSC6668 and N315). The *ccrB* gene (KU523873) of MPU SH 68 shared the highest identity (97 %) with the *ccrB2* gene of *S. aureus* strain JCSC1968 and *S. epidermidis* CS8. Thus, the nucleotide sequences of the studied *ccrAB* genes showed that they can not be assigned to known allotypes based on the cut-off value of 85 % identity. Our present data, together with previous reports, emphasize the great diversity of the SCC*mec* elements in *S. haemolyticus* (Bouchami et al. 2012; Hanssen and Ericson Sollid 2007; Pi et al. 2009; Urushibara et al. 2011; Zong and Lü 2010).

In conclusion, *S. haemolyticus* has been found to be an important source of the *mec* complex C and the *ccrC* complex, which are components of SCC*mec* type V. The analysis of *ccrA and ccrB* gene sequences of the *S. haemolitycus* strain (MPU SH 68) showed their high nucleotide sequence similarity to those found in *S. aureus* and *S. epidermidis* strains. Moreover, the new combination, i.e., *mec* complex B/*ccrAB*_ship_, was identified in coagulase-negative staphylococci. Our study underscores the great diversity of SCC*mec* structures in *S. haemolitycus* strains, as well as the importance of these bacteria as a reservoir of *mecA* genes.
